# Systemic analysis of the expression and prognostic significance of USP31 in endometrial cancer

**DOI:** 10.17305/bjbms.2022.8440

**Published:** 2023-05-01

**Authors:** Yuzhen Huang, Peng Jiang, Yuting Chen, Jinyu Wang, Rui Yuan

**Affiliations:** 1 Department of Gynecology, The First Affiliated Hospital of Chongqing Medical University, Chongqing, China; 2 College of Biomedical Engineering, Chongqing Medical University, Chongqing, China

**Keywords:** Endometrial cancer (EC), ubiquitin-specific protease 31 (USP31), biomarker, prognosis

## Abstract

Increasing evidence indicates that multiple mechanisms are involved in the metastasis and postoperative recurrence in patients with endometrial cancer (EC). Ubiquitin-specific protease 31 (USP31) has been studied in some human tumors, but its function remains unclear in EC. In this study, we tried to investigate the expression of USP31 in EC and its possible involvement in biological signaling pathways and define its predictive value for the prognosis. Data from The Cancer Genome Atlas (TCGA) and Genotype-Tissue Expression (GTEx) databases confirmed the difference in *USP31* expression between EC and normal endometrium. Specimens and clinical data of 259 patients with EC who underwent primary surgery at the First Affiliated Hospital of Chongqing Medical University were collected. The independent predictive value of USP31 for the prognosis of EC patients was determined by univariate and multivariate analyses. Kaplan–Meier analysis and receiver operating characteristic curves were used for confirming the ability of USP31 to predict the prognosis. Functional enrichment analyses were used for finding the hub genes associated with *USP31* and to predict the biological signaling pathways that might be involved. Our study confirms that EC patients with low expression of USP31 may have a worse prognosis. Functional annotations suggest that USP31 may participate in the mitogen-activated protein kinase signaling pathway, nuclear factor κB pathway, early 2 factor targets, and inflammatory response. USP31 may act as a promising biomarker for research in EC.

## Introduction

Endometrial cancer (EC) is one of the most common gynecological cancers, with a growing incidence worldwide [1]. Previously, the incidence was reported to be higher in high-income countries, but in recent years, the incidence in East Asia has not been negligible due to the improvement of quality of life and changes in the perception of fertility [2]. There is still no effective and personalized treatment for patients with recurrent and metastatic EC. Several studies have attempted to illustrate the mechanism of recurrence and metastasis in EC, but it remains unclear. It is reported that many mechanisms, such as epithelial mesenchymal transition (EMT), immune escape, and regulation of ubiquitination, may be involved in the process of recurrence and metastasis in EC [3–5]. It is of great significance to explore the causes and underlying molecular mechanisms to identify potential molecular biomarkers for early diagnosis, prevention, and personalized treatment.

Protein ubiquitination is an important mechanism for regulating the protein activity and levels under physiological conditions [6]. Ubiquitin-specific peptidases (USPs) are the main class of deubiquitinases that can remove ubiquitin modifications [7]. The USPs family has over 50 identified types to date [6]. They are found throughout the human body in various systems, and most of them are expressed in normal human tissues and tumor tissues. They have a variety of complex roles, such as participating in DNA damage repair, Wnt/β-catenin signaling, transforming growth factor β (TGF-β) pathway, c-Myc pathway, nuclear factor κB (NF-κB) pathway, p53 pathway, etc. [7]. Their expression and potential prognostic prediction values were discussed in different tumors. USP32 is expressed in primary ovarian cancer, especially in metastatic peritoneal tumors, and results in worse survival outcome [8]. USP22 may lead to poor prognosis in salivary adenoid cystic carcinoma via c-Myc pathway [9], whereas USP35 has been linked to an inhibition of the NF-κB pathway [10]. There are only a few studies on USP31, but all of them confirmed the importance of USP31 in tumor progression. The suppressed activity of USP31 may lead to cisplatin-induced apoptosis resistance in HeLa cell line [11]. Moreover, it is reported that USP31 may act as a suppressor in NF-κB pathway in sarcoma genesis [12]. By now, the functions of USP31 in EC remain unclear. Therefore, understanding the regulation and molecular function of USP31 may indicate the next potential therapeutic target and prognosis predictor for EC.

To achieve this aim, we analyzed the expression of USP31 and collected clinical data and specimens from 259 patients with EC in the first affiliated hospital of Chongqing Medical University to reveal the potential prognostic predictive value of USP31 by immunohistochemistry (IHC). We then attempted to analyze its potential molecular interaction network and role in biological process. Our findings may reveal the value of USP31 as a biomarker in predicting the prognosis of patients with EC, as well as provide new perspectives and inspiration for research into the role of USP31 in EC.

## Materials and methods

### Transcriptional expression profile

Gene Expression Profiling Interaction Analysis (GEPIA; http://gepia.cancer-pku.cn/) is a Web server for the analysis of RNA sequencing expression data from The Cancer Genome Atlas (TCGA) and Genotype-Tissue Expression (GTEx) databases [13]. We compared the expression of *USP31* between tumors and normal tissues in different tumors. Afterward, we compared the differential expressions of *USP31* in normal endometrial tissues and EC tissues. ANOVA test was used for defining the significance.

### The Human Protein Atlas

The Human Pathology Atlas project (HPA; https://www.proteinatlas.org) contains IHC data using a tissue microarray-based analysis on 44 different normal tissue types, and proteome analysis of 17 major cancer types [14]. Detailed information of tissue sections, such as intensity, location of the protein, and the respective cancer types, were available on HPA. Different protein expressions of USP31 in EC and in normal tissues were detected by IHC.

### Patients profile

With the patients’ consent, we collected pathological specimens and clinical data from 259 patients with EC who underwent primary surgery at the First Affiliated Hospital of Chongqing Medical University from January 2017 to June 2020. The data included the following: age, body mass index (BMI), pathological parameters, follow-up, prognosis outcome, etc. The following cases were excluded: 1) without standardized surgical treatment; 2) with others malignant tumors; 3) incomplete case data; 4) without follow-up after surgery. The details are shown in Figure S1.

### Immunohistochemistry staining and evaluation

All specimens were preserved in formalin and sent to the pathological laboratory of Chongqing Medical University for the next process. The process was coordinated with the same standards of pathological department [15]. Paraffin was used for fixing the specimens. Then the specimens were cut into 3–5 µm slices. Hematoxylin-eosin staining was used to determine the most heterogeneous part of the specimen which would represent the specimen. At the same time, 10 specimens of normal endometrium were collected (patients with uterine malformation) for the subsequent experiments.

The IHC for estrogen receptor α (ERα), progesterone receptor (PR), p53, Ki67, and USP31 were performed as follows. Slices were dried in 60 ^∘^C for 12 h and then deparaffinized using xylene. Epitopes were retrieved at a temperature of 100 ^∘^C for about 20 min, cooled down to 20 ^∘^C, and then bathed in 0.3% H_2_O_2_ solution in methanol for 5 min to block endogenous peroxidase activity. Slices were incubated with antibodies for at least 12 h at 4 ^∘^C: ER (SP1, in 1:50), PR (MX009, in 1:500), Ki67 (MX006, in 1:300), p53 (MX008, in 1:200) (Maixin Biotech, China), and USP31 (ab240543, abcam, 3–5 µg/mL). Secondary antibody incubation was performed using anti-mouse secondary antibody (Leica) for ERα, PR, p53, and Ki67 and anti-goat secondary antibody for USP31 (Jackson). 3,3’-diaminobenzidine tetrahydrochloride (DAB Substrate System, manufactured by DAKO) and hematoxylin were used for coloring the slices.

Two experienced pathologists evaluated the same slice and recorded the average percentage of positive-stained cells in five random microscopic fields (blind). Two results were considered consistent if the results differed ≤ 10%; otherwise, they would reevaluate the result and reach a consensus (unblind). The average percentage of two pathologists’ results represented the ultimate result of the slice. Cohen’s kappa was used to assess the agreement of the results between the two pathologists [16].

### Statistical analysis

Referring to other studies, we set the cutoff value of 4 factors as follows: ERα 5%; PR 5%; Ki67 40% [15, 17, 18], while the p53 was classified as normal or abnormal. Based on this, we defined the patients’ groups as high or low expression groups. Patients with the percentage > 50% of USP31 positive-stained cells were defined as the high expression group and those with the percentage ≤ 50% were defined as the low expression group. Kaplan–Meier analyses were utilized to identify the differences of recurrence-free survival (RFS) and overall survival (OS) between the two groups. The proportional hazard model (COX regression model) was used for analysis of the common factors that would affect the prognosis of patients with EC, such as age, BMI, cervical stromal invasion, myometrial invasion, and expression levels of ERα, PR, p53, and Ki67. Among them, age and BMI were both treated as continuous variables, while others were categorical variables. Two-sided *P* value < 0.05 was considered to have significance. The remaining factors were further analyzed by multivariate analysis. We chose the factors with a *P* value < 0.05 in multivariate analysis for constructing the logistic model and tested its accuracy by the receiver operating characteristic (ROC) curve. SPSS 26.0 was used for statistical analysis.

### Protein–protein interaction network construction

Protein–protein interaction (PPI) network was constructed by using the Biological General Repository for Interaction Datasets (BioGRID 4.4; https://thebiogrid.org/). BioGRID is a public database that archives and disseminates genetic and protein interaction data from model organisms and humans [19]. A minimum of one evidence of the interaction was considered significant.

### Functional annotations

The hub genes in PPI network would undergo Gene Ontology (GO) enrichment and Kyoto Encyclopedia of Genes and Genomes (KEGG) enrichment by using Database for Annotation, Visualization, and Integrated Discovery (DAVID 2021; https://david.ncifcrf.gov/) [20]. False discovery rate (FDR) < 0.05 was considered statistically significant. The results were shown as bar plots. Gene set enrichment analysis (GSEA) was used for the prediction of the potential hallmarks of *USP31* using the TCGA transcriptional sequence database. A permutation test with 1000 times was used for identifying the significantly changed pathways. Adjusted *P* value < 0.01 and FDR values < 0.25 were considered significant. In addition, the top 100 highly correlated genes were identified. Graphical plotting was conducted by using R software 4.1.0.

### Immune infiltration analysis

Tumor Immune Estimation Resource (TIMER) is a Web server for comprehensive analysis of tumor-infiltration immune cells of selected hub genes [21]. An integrated repository portal for tumor–immune system interactions (TISIDB; http://cis.hku.hk/TISIDB/index.php) was utilized to examine the interactions of tumor and immune system in 28 types of tumor-infiltrating lymphocytes (TILs) across human cancers [22]. The relative abundance of TILs was inferred by using gene set variation analysis based on *USP31* expression profile. Spearman’s test was used to measure the correlations between *USP31* and TILs. All tests were two-sided and *P* values < 0.05 were considered significant.

## Results

### Differential expression of USP31

**Figure 1.**
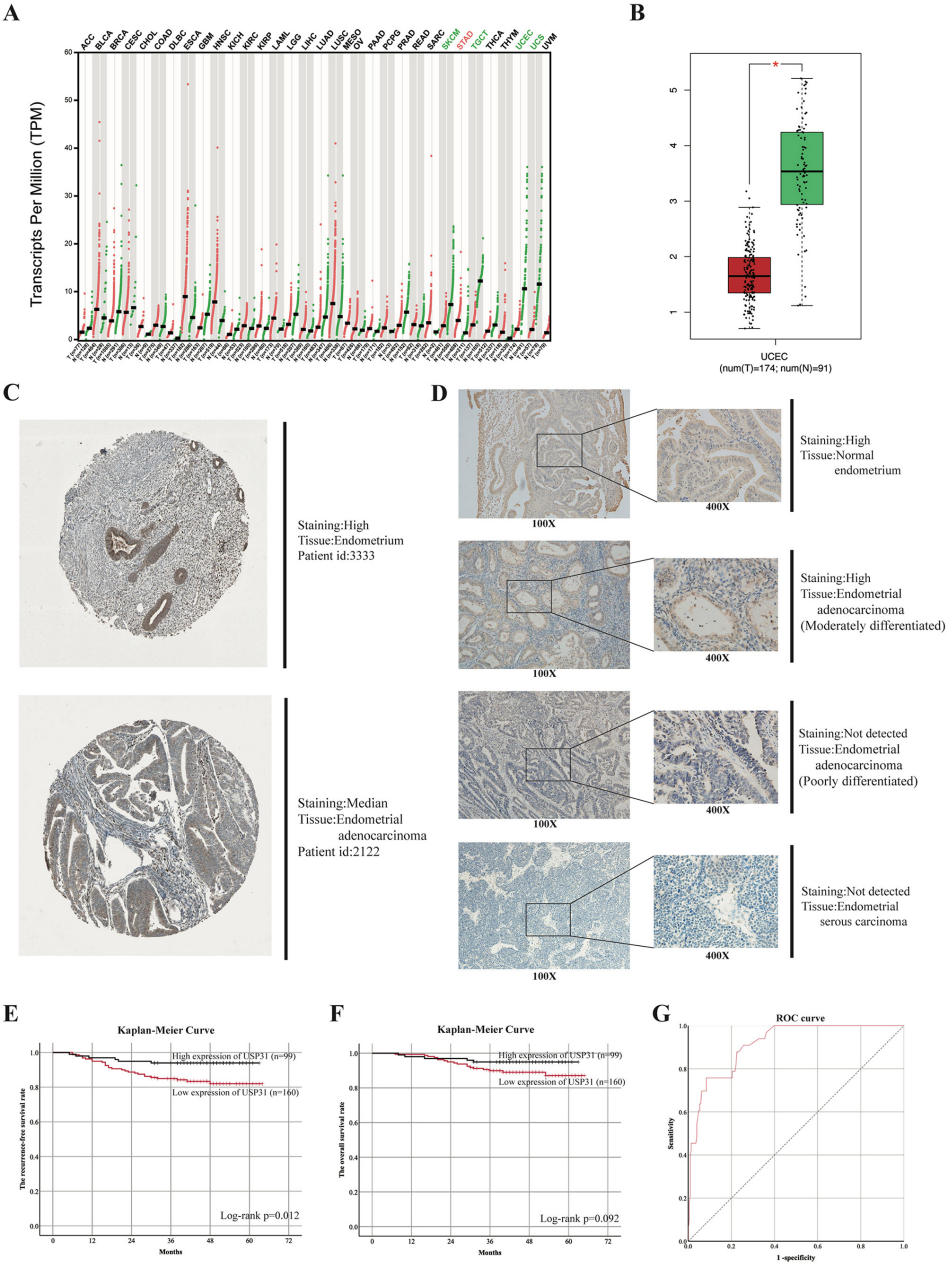
**Differential expressions of USP31 affect the prognosis of patients with EC.** (A) The differential expression of *USP31* in various tumors compared with their normal tissues. Uterine corpus endometrial carcinoma shows significant difference; (B) The difference in expression of *USP31* between 174 tumor samples and 91 normal samples from TCGA and GTEx databases (*P* < 0.01); (C) The diversity of USP31 expression in endometrial tissue and EC from HPA (Endometrium, patients id: 3333, high-staining; Endometrial adenocarcinoma, patient id: 2122, median-staining); (D) The differential staining of USP31 in CQMU cohort. Patients in CQMU cohort were divided into two groups based on the staining level of USP31: high expression group (*n* ═ 99) and low expression group (*n* ═ 160); (E) Kaplan–Meier curve of USP31 grouping for recurrence-free survival (*P* ═ 0.012); (F) Kaplan–Meier curve of USP31 grouping for overall survival (*P* ═ 0.092); (G) ROC curve was generated to validate the ability of the logistic model to predict recurrence of patients with EC after surgery (AUC index ═ 0.915). EC: Endometrial cancer; CQMU: Chongqing Medical University; ROC: Receiver operating characteristic; AUC: Area under the curve; USP31: Ubiquitin-specific protease 31; TCGA: The Cancer Genome Atlas; GTEx: Genotype-Tissue Expression; UCEC: Uterine corpus endometrial carcinoma; HPA: The Human Pathology Atlas.

Figure 1A shows the different mRNA expressions of *USP31* in various tumors and their corresponding normal tissues. Based on 174 tumor samples and 91 normal samples from TCGA and GTEx database, it is obvious that the difference in mRNA expressions between EC and normal endometrial tissues was significant, proved by ANOVA test (*P* value < 0.01) (Figure 1B). Moreover, immunohistology from the HPA database revealed the diversity of protein expressions of USP31 in endometrium and EC (Figure 1C) (Endometrium, patient id 3333; Endometrial adenocarcinoma, patient id: 2122).

### Clinical characteristics and pathological data in two groups

As we have mentioned in the previous part, we divided 259 patients into two groups based on IHC results: high USP31 expression (*n* ═ 160) and low USP31 expression (*n* ═ 99). Cohen’s kappa index was 0.753, which was considered substantial. Figure 1D shows the different expressions of USP31 in our cohort. The clinical characteristics of the two groups are recorded in Table 1. Age (*P* ═ 0.768), FIGO staging (*P* ═ 0.778), cervical stromal invasion (*P* ═ 0.958), myometrial invasion (*P* ═ 0.944), grade (*P* ═ 0.122), p53 expression (*P* ═ 0.770), Ki67 expression (*P* ═ 0.367), ERα expression (*P* ═ 0.386), and PR expression (*P* ═ 0.098) showed no statistical significance between the groups. Patients in the two groups had some differences in BMI (*P* ═ 0.019). There were also significant differences in prognostic outcomes (Table 2) The median RFS of the high expression group was 49 months, whereas the median RFS of low expression was 43 months. Patients in the high expression group had median OS of 49 months, which was greater than 46 months in the low expression group. The low expression group had a higher percentage of recurrence and death than the high expression group. Kaplan–Meier analysis was used for identifying the difference of RFS and OS between the two groups. The results indicated that RFS rate obtained *P* ═ 0.012, which was considered to have statistical significance. The Kaplan–Meier curves are shown in Figure 1E and 1F.

**Table 1:** Clinicopathological characteristics in relation to USP31 expression level

	**CQMU Cohort (*n* ═ 259)**		
**Characteristics**	**High USP31 expression (*n* ═ 99)**	**Low USP31 expression (*n* ═ 160)**	***P* value**
*Age (years)*			0.768
Mean ± SD	52.59 ± 10.27	52.94 ± 8.96	
Median (range)	52 (26–78)	52 (24–81)	
*BMI (kg/m^2^)*			0.019
Mean ± SD	24.05 ± 3.81	25.15 ± 3.50	
Median (range)	23.83 (16.35–39.30)	24.93 (18.03–39.30)	
*FIGO Staging (n, %)*			0.778
I	79, 79.8%	133, 83.1%	
II	9, 9.1%	13, 8.1%	
III	11, 11.1%	14, 8.8%	
*Cervical stromal invasion (n, %)*			0.958
Yes	14, 14.1%	23, 14.4%	
No	85, 85.9%	137, 85.6%	
*Myometrial invasion (n, %)*			0.944
Yes	27, 27.3%	43, 26.9%	
No	72, 72.7%	117, 73.1%	
*Grade (n, %)*			0.122
G1	49, 49.5%	62, 38.8%	
G2	36, 36.4%	61, 38.1%	
G3	14, 14.1%	37, 23.1%	
*ERα (n, %)*			0.386
High	89, 89.9%	138, 86.3%	
Low	10, 10.1%	22, 13.8%	
*PR (n, %)*			0.098
High	92, 92.9%	138, 86.3%	
Low	7, 7.1%	22, 13.8%	
*p53 (n, %)*			0.787
Normal	69, 69.7%	97, 60.6%	
Abnormal	30, 30.3%	63, 39.4%	
*Ki67 (n, %)*			0.564
High	35, 35.4%	51, 31.9%	
Low	64, 64.6%	109, 68.1%	

CQMU: Chongqing Medical University; SD: Standard deviation; BMI: Body mass index; FIGO: International Federation of Gynecology and Obstetrics; ERα: Estrogen receptor α; PR: Progesterone receptor; USP31: Ubiquitin-specific protease 31.

**Table 2:** Clinical outcome in relation to USP31 expression level

	**CQMU Cohort (*n* ═ 259)**		
**Characteristics**	**High USP31 expression (*n* ═ 99)**	**Low USP31 expression (*n* ═ 160)**	***P* value**
*Recurrence (n, %)*			0.011
Yes	6, 6.1%	27, 16.9%	
No	93, 93.9%	133, 83.1%	
*Death (n, %)*			0.088
Yes	5, 5.1%	18, 11.3%	
No	94, 94.9%	142, 88.8%	
*RFS (months)*			0.030
Median (range)	49 (5–63)	43 (6–64)	
*OS (months)*			0.170
Median (range)	49 (8–63)	46 (7–65)	

CQMU: Chongqing Medical University; RFS: Recurrence-free survival; OS: Overall survival; USP31: Ubiquitin-specific protease 31.

### COX regression analyses and ROC curve

We performed a univariate analysis of common factors affecting postoperative recurrence and death in patients with EC included in this study, and the results were presented in Tables S1 and S2. Statistically significant factors were then subjected to multivariate analysis. The results suggested that FIGO staging, grade, ERα, p53, Ki67, and USP31 were statistically significant in the COX multivariate regression analysis for predicting postoperative recurrence in EC patients (Table 3). In the COX regression analysis for postoperative death, USP31 showed no statistical significance. Although the *P* value of USP31 did not support its value in predicting postoperative death in EC patients, USP31 still obtained a small *P* value of 0.102. Accordingly, we established a formula (based on the sum of hazard ratio values of statistically significant variables) to predict the risk of recurrence in EC patients: [1.531×FIGO II or 9.335×FIGO III] FIGO Staging (ref. I) + [1.621×G2 or 4.768×G3] Grade (ref. G1) + 3.844×ERα (ref. High) + 2.528×p53 (ref. Normal) + 2.730×Ki67 (ref. Low) + 0.342×USP31 (ref. Low) (Assigning a value of 1 if it does not belong to the reference category), which had a relatively good accuracy confirmed by ROC curve. The area under the curve of ROC curve was 0.915 (Figure 1G).

**Table 3:** Multivariate Cox logistic regression analysis of recurrence-free survival

**Covariates**	**HR**	**95% CI**	***P* value**
Age	1.009	0.969–1.052	0.654
Cervical stromal invasion (ref. No)	1.552	0.303–7.945	0.598
Myometrial invasion (ref. No)	1.360	0.478–3.874	0.564
*FIGO Staging (ref. I)*			0.007
FIGO II	1.531	0.178–13.194	0.699
FIGO III	9.335	1.685–51.704	0.011
*Grade (ref. G1)*			0.031
G2	1.621	0.514–5.116	0.410
G3	4.768	1.412–16.105	0.012
ERα (ref. High)	3.884	1.442–10.392	0.008
PR (ref. High)	1.307	0.542–3.152	0.551
p53 (ref. Normal)	2.528	1.045–6.114	0.040
Ki67 (ref. Low)	2.730	1.236–6.030	0.013
USP31 (ref. Low)	0.342	0.131–0.890	0.028

FIGO: International Federation of Gynecology and Obstetrics; ERα: Estrogen receptor α; PR: Progesterone receptor; USP31: Ubiquitin-specific protease 31; HR: Hazard ratio; CI: Confidence interval.

### Functional annotations and predicted signaling pathway

**Figure 2.**
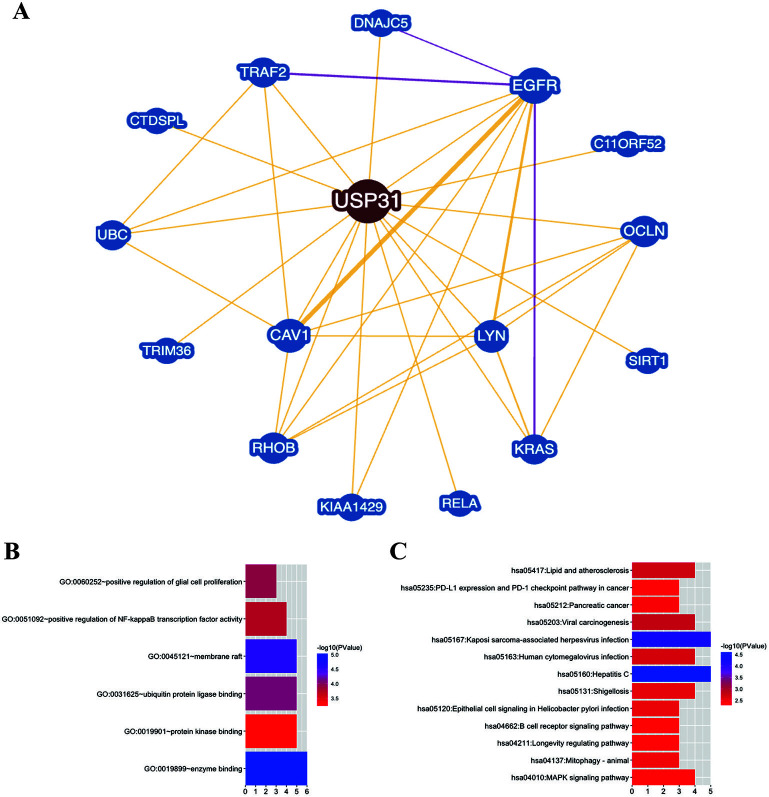
**Functional annotations and predicted signaling pathways.** (A) The protein–protein interaction network was constructed. A network of *USP31* and its interacted genes was set up visually; (B) Functional GO enrichment analysis of a total of 16 involved hub genes. The result is shown as bar plot. Significant genes were significantly involved in enzyme binding, protein kinase binding, ubiquitin protein ligase binding, membrane raft, positive regulation of NF-κB transcription factor activity, and positive regulation of glial cell proliferation; (C) Functional KEGG enrichment analysis of a total of 16 involved hub genes. The result is shown as bar plot. Significant genes were significantly involved in MAPK signaling pathway, mitophagy-animal, longevity regulating pathway, B cell receptor signaling pathway, epithelial cell signaling in *Helicobacter Pylori* infection, shigellosis, hepatitis C, human cytomegalovirus infection, Kaposi sarcoma-associated herpesvirus infection, viral carcinogenesis, pancreatic cancer, PD-L1 expression and PD-1 checkpoint pathway in cancer and lipid metablism and atherosclerosis. GO: Gene Ontology; KEGG: Kyoto Encyclopedia of Genes and Genomes; USP31: Ubiquitin-specific protease 31; NF-κB: Nuclear factor κB; MAPK: Mitogen-activated protein kinase; PD-1: Programmed death protein 1; PD-L1: Programmed death-ligand 1.

Figure 2A shows the PPI network. We had collected 16 hub genes in total. GO enrichment showed that the PPI network might be involved in enzyme binding, protein kinase binding, ubiquitin protein ligase binding membrane raft, positive regulation of NF-κB transcription factor activity, and positive regulation of glial cell proliferation (Figure 2B). KEGG enrichment suggested that hub genes played an important role in programmed death-ligand 1 (PD-L1) expression and PD-L1 checkpoint pathway in cancer, mitogen-activated protein kinase (MAPK) signaling pathway, and B cell receptor signaling pathway (Figure 2C).

### Significant genes and pathways obtained by GSEA

**Figure 3.**
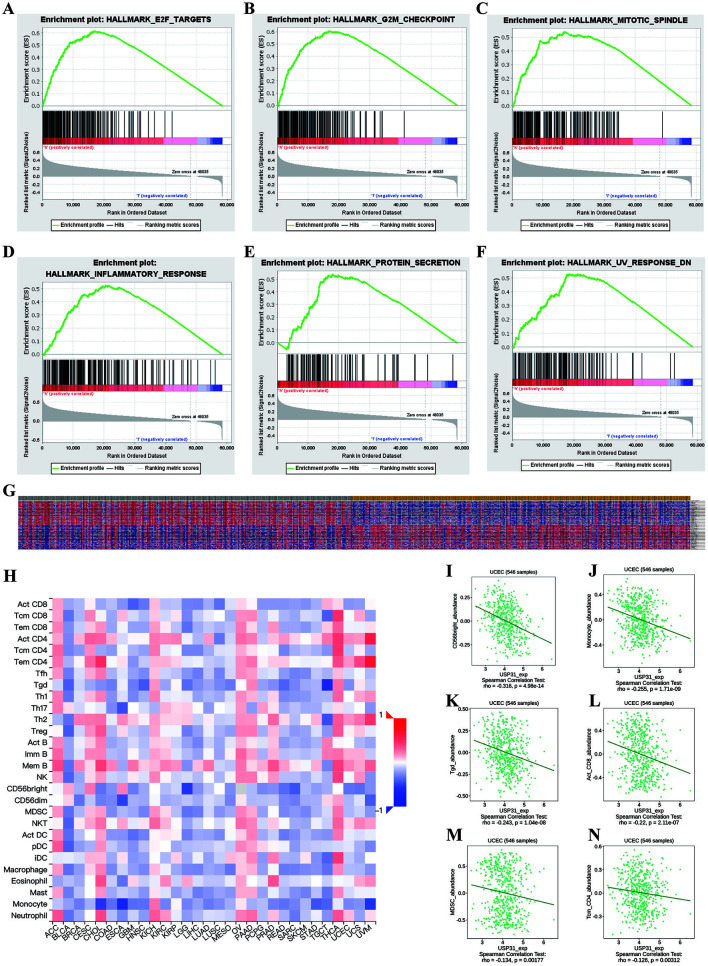
**Gene set enrichment analysis (GSEA) and immune analysis.** (A)–(F) The most involved significant pathways included E2F targets, G2M checkpoint, mitotic spindle, inflammatory response, protein secretion, and UV response; (G) Transcriptional expression profiles of 100 significant genes with positive and negative correlation with *USP31* were performed in a heat map; (H) A heat map shows the correlations of expression of *USP31* and 28 TILs across various human cancers; (I)–(N) The most significant TILs associated with *USP31* in endometrial cancer, included CD56 bright natural killer cells (CD56bright) (rho ═ −0.316, *P* < 0.05), monocytes (rho ═ −0.225, *P* < 0.05), gamma delta T cells (Tgd) (rho ═ −0.243, *P* < 0.05), activated CD8 T cells (Act CD8) (rho ═ −0.22, *P* < 0.05), myeloid derived suppressor cells (MDSC) (rho ═ −0.134, *P* < 0.05) and central memory CD4 T cells (Tcm CD4) (rho ═ −0.126, *P* < 0.05). TIL: Tumor-infiltrating lymphocytes; USP31: Ubiquitin-specific protease 31; E2F: Early 2 factor; UV: Ultraviolet.

Hallmarks analysis indicated that *USP31* might be involved in early 2 factor (E2F) targets, G2-M checkpoint, mitotic spindle, inflammatory response, protein secretion, and ultraviolet response (Figure 3A–3F). By using GSEA analysis, we obtained the top 100 correlated genes for *USP31* (Figure 3G). The details are shown in Figure S2.

### Correlation of USP31 expression and immune infiltration

Based on the results from TIMER, with the growing expression of *USP31*, the infiltration level of neutrophils increases slightly. However, changes in other immune cells were not statistically significant, which included B cells, CD8+ T cells, macrophages, etc. Details are shown in Figure S3. However, the results of GSEA analysis suggested that inflammatory responses might also play a role in tumor progression associated with *USP31*. Therefore, we utilized TISIDB to determine the correlation between *USP31* and TILs in different human cancers (Figure 3H). Several TILs were negatively correlated with *USP31* expression in many human cancers. At the same time, we found a significant correlation of *USP31* with some of the 28 types of TILs with more detailed classification in EC. *USP31* significantly correlated with the decrease of CD56 bright natural killer cells (rho ═ –0.316, *P* < 0.05), monocytes (rho ═ –0.225, *P* < 0.05), gamma delta T cells (rho ═ –0.243, *P* < 0.05), activated CD8+ T cells (rho ═ –0.22, *P* < 0.05), myeloid derived suppressor cells (rho ═ –0.134, *P* < 0.05), central memory CD4+T cells (rho ═ –0.126, *P* < 0.05). The results are shown in Figure 3I–3N.

## Discussion

Many studies had confirmed that molecular mechanisms such as EMT and immune escape widely influence tumor metastasis and recurrence, including EC. Also, the USPs family plays an important role in tumor metastasis and recurrence. Many members of the USPs family have been mentioned in many studies for their effects on the tumor process, as well as for their effects in tumor recurrence and drug resistance. However, there were few studies on USP31, especially for EC. Using data from TCGA and GTEx, we found that the expression of USP31 was significantly reduced in EC. Specimens collected in our cohort and long-term follow-up data confirmed that patients with low expression of USP31 had a poorer prognosis, especially reflected in RFS. USP31 could be identified as an independent biomarker in predicting postoperative recurrence in patients with EC. In addition, functional enrichment and GSEA analysis suggested that USP31 might influence the prognosis of patients with EC through several important pathways in tumors, such as MAPK signaling pathway, NF-κB pathway, E2F targets, and inflammatory response.

MAPK signaling pathway has been proven to be highly involved in several types of human cancers [23]. It also has a great impact on the migration and invasion of tumors. For example, taraxastane may inhibit the migration and invasion of human cervical cancer by regulating MAPK signaling [24]. Research also revealed that MAPK signaling pathway might play an important role in drug resistance [25]. Activation of MAPK signaling led to enzalutamide resistance in prostate cancer [26].

NF-κB pathway was increasingly recognized as a crucial pathway in cancer progression [27]. Promoting NF-κB pathway would drive breast cancer metastasis and lead to immune suppression [28]. Due to the discovery that NF-kB inhibition sensitized EC cells to standard EC chemotherapy (paclitaxel/carboplatin) toxicity, NF-κB pathway was proposed as a new target for EC treatment [29]. These studies have confirmed the significant functions of NF-κB pathway in tumor progression, which include metastasis and drug resistance that could highly affect the prognosis.

Missed expression of E2F transcriptional targets might lead to broken cell cycle [30]. Researchers have identified E2F targets as a potential clinically actionable target [31]. The upregulation of E2F pathway would significantly drive melanoma metastasis [32]. Apparently, E2F target are of great significance in tumor progression.

The immune system has crucial roles in cancer development and treatment [33]. In the tumor microenvironment, various inflammatory cells were present, which include neutrophils, T lymphocytes, etc. [34]. Inflammatory microenvironment and changes in genes might lead to tumor progression and metastasis [35]. Research showed that inflammation enhances cancer progression in pancreatic tumor in part by facilitating EMT and entry into the circulation [36]. In addition, the treatment of mice with anti-inflammatory agents suppressed the senescence-associated inflammatory response and prevented p53-deficient mice carcinogenesis [37].

Many studies have attempted to predict the prognosis of EC in multiple ways. The role of biomarker has received a lot of attention. TCGA had proposed four prognostic groups [38]. Probing the status of specific molecules (e.g., CTNNB1, etc.) could classify patients into four types: POLE-mutated/ultramutated (POLEmt), microsatellite-instable/hypermutated (MSI), copy-number-high/p53-mutated (p53mt), and no specific molecular profile (NSMP), corresponding to different prognoses. However, gene sequencing is not yet widely available in many cases, especially in developing countries due to the high cost. Therefore, attempts had been made to find the alternative methods, such as postoperative IHC, to indicate the prognosis of EC patients. Studies had focused on the role of non-coding RNA (ncRNA) for the same purpose. Also, ncRNA dysregulation has been found to affect the prognosis of EC through multiple molecular pathways [39, 40]. In parallel, serum metabolomic assays have been proposed for differentiating EC from other conditions with an advantage of minimal invasiveness [41]. It would also be interesting to further explore its role in predicting prognosis. There is no report on USP31 affecting the prognosis of patients with EC. However, it is noteworthy that some studies have started to focus on the role of USP31 in tumor progression. In our study, multivariate analysis confirmed USP31 as an independent influencing factor. This also reminded us that the importance of molecular indicators should not be neglected in clinical applications. Certainly, we cannot ignore the role of traditional pathological parameters, which still serve as the main part of current guidelines (such as American National Comprehensive Cancer Network [42] and ESGO/ESTRO/ESP [43]). It had been suggested that lymphovascular space invasion used in conjunction with TCGA signature in the MSI group allowed for more detailed stratification of patients [44].

It is highly likely that USP31 will become a new important biomarker of EC. Our cohort confirmed that low USP31 expression was associated with poorer prognosis in EC. This suggested that USP31 may play a suppressor role in tumor progression. Therefore, more aggressive adjuvant therapy and more frequent follow-up are necessary for patients with low USP31 expression (or not detected) to achieve better outcome. To explore the molecular biological mechanism behind USP31, we obtained hub genes by constructing a PPI network and performed functional enrichment analysis. Moreover, based on data from TCGA, we performed a GSEA analysis to determine the potential hallmarks which UPS31 may highly involve. TISIDB analysis suggested that the expression of *USP31* might lead to the decrease of specific TILs. It may be one of its ways to suppress tumor metastasis by regulating the immune response as well, which undoubtedly corroborated the important role of USP31 in tumors.

This study has several limitations. First, the *P* value of USP31 suggested no statistical significance in the multivariate analysis for OS in EC patients, which might be due to the limited number of deaths. At the same time, the different choices of therapy after recurrence, which we have not discussed in this study, also affected the OS of patients, but it still obtained a small *P* value of 0.141. Secondly, although we identified the potential signaling pathways in which USP31 might be involved, we did not elaborate the specific molecular mechanisms behind them. However, this study still provided future researchers with ideas to investigate the role of USP31 in EC.

## Conclusion

Our study confirmed the differences in USP31 expressions between EC and normal endometrium. EC patients with low USP31 expression had worse postoperative prognosis, which was associated with many important pathways in tumor. Study confirmed the predictive value of USP31 for postoperative recurrence in EC patients. USP31 is a promising biomarker for EC. This study provided a new and promising insight for subsequent studies. Further studies are required to focus on the potential molecular mechanisms behind USP31 and its clinical applications.

## Acknowledgments

We would like to thank the department of Gynecology of the First Affiliated Hospital of Chongqing Medical University for its support.

## Supplemental Data

Supplementary data are available at the following link: https://www.bjbms.org/ojs/index.php/bjbms/article/view/8440/2665.

Detailed Figure S2 is available at the following link: https://www.bjbms.org/article_images/8440_Figure_S2.pdf.
